# Myocarditis: imaging up to date

**DOI:** 10.1007/s11547-020-01279-8

**Published:** 2020-10-06

**Authors:** Carlo Liguori, Davide Farina, Filippo Vaccher, Giovanni Ferrandino, Davide Bellini, Iacopo Carbone

**Affiliations:** 1 Radiology Unit, Ospedale del Mare- A.S.LNa1-Centro, 80147, Naples, Italy; 2 Department of Medical and Surgical Specialties, Radiological Sciences, and Public Health, University of Brescia - ASST Spedali Civili of Brescia, Brescia, Italy; 3 Department of Precision Medicine, University of Campania Luigi Vanvitelli, 80138 Naples, Italy; 4 Department of Radiological, Oncological and Pathological Sciences, “Sapienza” University of Rome, I.C.O.T. Hospital, Via Franco Faggiana1668, 04100 Latina, LT Italy

**Keywords:** Myocarditis, Myocardial inflammation, Cardiac MR, Magnetic resonance imaging

## Abstract

Myocarditis is an inflammatory disease of the heart muscle, diagnosed by histological, immunological, and immunohistochemical criteria. Endomyocardial biopsy represents the diagnostic gold standard for its diagnosis but is infrequently used. Due to its noninvasive ability to detect the presence of myocardial edema, hyperemia and necrosis/fibrosis, Cardiac MR imaging is routinely used in the clinical practice for the diagnosis of acute myocarditis. Recently pixel-wise mapping of T1 and T2 relaxation time have been introduced into the clinical Cardiac MR protocol increasing its accuracy. Our paper will review the role of MR imaging in the diagnosis of acute myocarditis.

## Introduction

In 1995 the World Health Organization (WHO)/International Society and Federation of Cardiology (ISFC) defined myocarditis as an inflammatory disease of the heart muscle, diagnosed by histological, immunological, and immunohistochemical criteria [1].

Myocarditis diagnosis is often challenging because of the heterogeneity of clinical presentations. The real incidence of myocarditis is difficult to work out as endomyocardial biopsy (EMB), the diagnostic gold standard, is employed infrequently [1–3].

Several published studies report a highly variable autopsy prevalence of myocarditis (2–42%).

Thanks to its unique ability to directly image myocardial necrosis, fibrosis and edema, cardiac magnetic resonance (CMR) is now considered the first tool for noninvasive assessment of patients with suspected myocarditis.

CMR is also useful for monitoring disease activity under treatment [4].

Myocarditis clinical resolution is often spontaneous in patients presenting with mild symptoms, even if in presence of minimal ventricular dysfunction.

However, in up to 30% of cases, biopsy-proven myocarditis can progress to dilated cardiomyopathy (DCM) [5].

CMR plays a role in the follow-up of such cases to detect the progression toward a dilatative phenotype [4].

The underlying etiology determines a patient prognosis variation.

In several myocarditis forms, a symptomatic treatment is sufficient but immunohistochemical and molecular biological analysis of EMB is in other cases vital to identify subjects needing an appropriate therapy [5].

## Pathophysiology of myocardial inflammation

Viral infections and post-viral immune-mediated responses are commonly implicated in heart muscle inflammation.

Molecular techniques, mainly reverse transcription-polymerase chain reaction (RT-PCR) amplification, suggest that the spectrum of most frequently detected viruses is constituted by: enterovirus, adenovirus, influenza viruses, human herpesvirus-6 (HHV-6), Epstein-Barr virus, cytomegalovirus, hepatitis C virus, parvovirus B19 and

Coronavirus (Sars-Cov2 real incidence is still unclear), reported in the myocardium of patients with myocarditis and DCM [5].

Moreover, heart inflammation can be triggered by non-viral infections like as Borrelia burgdorferi (Lyme disease), Corynebacterium diphtheriae, or Trypanosoma cruzi (Chagas disease) [6].

Apart from infectious agents, several medications like antipsychotics (e.g., clozapine), antibiotics (penicillin, ampicillin, sulfonamides, tetracyclines), and anti-inflammatory (e.g., mesalamine), as well as toxic agents (like drugs used illicitly), can induce hypersensitivity eosinophilic myocarditis, which is usually reversible after withdrawal of the causative agent. Eosinophilic-lymphocytic myocarditis can also occur after smallpox vaccination [7].

Autoimmune diseases with systemic implication such as Churg-Strauss syndrome or hypereosinophilic syndrome (Loeffler’s disease) can be associated with eosinophilic myocarditis. Sarcoidosis and giant cell myocarditis are rare causes of inflammatory myocardial disease [3], (Table 1). Myocarditis may evolve in dilated cardiomyopathy (DCM), the most frequent reason for heart transplantation [8, 9].

**Table 1:** Etiological causes of myocarditis in relation to literature data

Infectious aetiologies (29%)		Non-infectious aetiologies (71%)	
Viral agents (28%) Adenoviruses Enteroviruses (coxsackievirus) Herpesviruses (Human Herpesviruses 6, Epstein-Barr virus) Hepatitis C virus HIV Influenza A Parovirus B19 (28%) Coronavirus (Sars-CoV2)	Bacterial agents (< 1%) Borrelia species Mycobacterium species Mycoplasma Pneumoniae Streptococcal species Treponema pallidum	Toxins (< 1%) Anthracyclines Cocaine Interleukin-2 Alcohol	Autoreactive Myocarditis (53%)
			Immunological Syndromes (< 2%) Churg-Strauss syndrome Diabetes mellitus Inflammatory bowel disease Giant cell myocarditis Granulomatosis with polyangiitis (Wegener granulomatosis) Sarcoidosis Systemic lupus erythematosus Takayasu arteritis Thyrotoxicosis
		Hypersensitivity (< 1%) Cephalosporins Dogoxin Diuretics Dobutamine Sulfonamides Tricyclic antidepressant	
	Fungal agents (< 1%) Aspergillus species Candida species Coccidioides species Cryptococcus species Histoplasma species		
Parasitic agents (< 1%) Larva migrans Schistosomiasis			
	Protozoal agents (< 1%) Trypanosoma Cruzi (Chagas disease)	Rejection (1%) After heart transplantation (1%) After stem cell transplantation	
			Other DCM patients (16%)

Human myocarditis pathophysiology is not completely understood. Murine models of enteroviral myocarditis suggest that the course of viral myocarditis is characterized by 3 phases, which might be simplified as follows [10]:

- The entry of the virus into the myocytes, mediated through a specific receptor is responsible for acute cell injury, induced by virus replication leading to necrosis, exposure of intracellular antigens like cardiac myosin and activation of the host’s immune system, which is characterized by the invasion of natural killer cells and macrophages followed by T lymphocytes. Acute phase covers only few days.
- Autoimmune reactions characterize the second phase, which may last for few days or protract up to several months. Activation is triggered by virus-specific T lymphocytes, which may target the host’s organs by molecular mimicry. Cardiac damage is aggravated by two events: cytokine activation (tumor necrosis factor [TNF]-alpha, interleukin [IL]-1/6) and antibodies to viral and cardiac proteins, leading to a cardiac contractile function impairment.
- The third (chronic) phase, not necessarily developed, is related to the persistence of autoimmune processes (regardless of the detection of the virus genome in the myocardium) and is the substrate for myocardial remodeling and DCM development [11].

In specific scenarios, non-infectious agents (drugs, toxins, etc.) damage myocytes directly or indirectly, causing the exposure of normally hidden antigens to the immune system with consequent activation of cross- or autoreactive T-cells and autoantibodies leading to myocardial damage.

## Clinical presentation

The disease, which can affect individuals of all ages, although it is more frequent in young people, has several clinical manifestations [5]:

- Asymptomatic forms.
- Acute forms, which resolve in about 50% of cases within 2–4 weeks: patient develops dyspnea or orthopnea, palpitations, effort intolerance/malaise, heart failure, chest pain with or without cardiac troponin I or T release and has unobstructed coronary arteries at coronary angiography. A pleuritic chest pain may be present in case of concomitant pericarditis. Palpitation, syncope or aborted sudden death due to unexplained new-onset atrial or ventricular tachy- or bradyarrhythmias can be observed. In the case of viral agents, a respiratory or gastrointestinal syndrome, with or without increased systemic inflammatory markers and fever, may precede (days or weeks) the clinical onset of cardiac signs and symptoms.
- Fulminant forms, presenting with unexplained acute heart failure.
- Chronic forms (about 25% of myocarditis) manifest with persistent cardiac dysfunction and in 12–25% may progress to end-stage inflammatory DCM. In these cases, patients present with symptoms of chronic or acute heart failure; more severe forms meet the indications for heart transplantation [12].

## CMR targets of myocarditis

CMR is able to identify 3 diagnostic targets during an acute inflammatory process of the myocardium: edema, hyperemia, and necrosis or fibrosis (Table 2). These 3 targets were proposed for the diagnosis of acute myocarditis by the first consensus on CMR in Myocardial Inflammation in 2009, the “Lake Louise Criteria” (LLC) [13].

**Table 2:** CMR targets, sequences and diagnostic criteria

Targets	Sequences	Diagnostic criteria
Myocardial edema^a^	T2-weighted imaging	Regional high T2 SI
		Global T2 SI ratio ≥ 2.0 in T2W CMR images
	T2-mapping	Regional or global increase of myocardial T2 relaxation time
Hyperemia	T1-weighted imaging (EGE)	SI ratio myocardium/skeletal muscle (EGE ratio) of ≥ 4.0 in EGE images
	T1-mapping	Regional or global increase of native myocardial T1 relaxation time or ECV
Necrosis/fibrosis	T1-weighted imaging (LGE)	Areas with high SI in a nonischemic distribution pattern in LGE images
	T1-mapping	Regional or global increase of native myocardial T1 relaxation time or ECV

*SI* signal intensity, *CMR* cardiac magnetic resonance, *EGE* early gadolinium enhancement, *LGE* late gadolinium enhancement, *ECV* extracellular volume

^a^Native T1 and ECV are also sensitive to myocardial inflammation and edema

Since their introduction the LLC were used by the majority of centers in the world in their daily clinical practice and have changed the clinical management of patients with a suspect of acute myocarditis [13].

Edema is the hallmark of inflammation in all soft tissues. It is a physiological response triggered by damage to living tissues mediated by several molecules including serotonin, bradykinin and prostaglandins. More in detail, in the setting of myocarditis, edema results from an imbalance between microvascular filtration, induced by microvascular endothelial barrier dysfunction, and lymphatics fluid removal; it is both intracellular and interstitial and can persist for several months [14].

On CMR, the increased tissue water content causes prolongation of both T1w and T2w relaxation times.

Edema can be assessed with traditional T2-weighted imaging and by means of T2 mapping techniques. Black-blood spin echo sequences (typically STIR) exploit T2 and T1 changes in myocardial edema with good diagnostic accuracy. Using triple inversion recovery techniques, fat suppression is improved, with signal-to-noise reduction as the principal drawback. T2-prepared steady-state free precession (SSFP)–based bright blood sequences could be considered a valid alternative [15]. On STIR T2-w images edema can be detected qualitatively but should always be assessed with T2 ratio: comparing the signal of the entire myocardium with the intensity of skeletal muscles on the same image. A ratio greater than 1.9 is considered abnormal.

T2 and T1 mapping allow quantification of myocardial edema, offering also the significant advantage of not requiring contrast agent administration.

Hyperemia is the first stage of the inflammatory response, a process characterized by a change in blood flow in the damaged area. Capillaries dilate increasing blood flow into the tissue, vascular permeability increases as well allowing fluid, proteins, and white blood cells to migrate out of the vessels. On CMR, this mechanism can be evaluated using the early gadolinium enhancement (EGE) technique, which consists in measuring the early contrast uptake of the myocardium acquiring T1w images within the first minutes after the administration of gadolinium-based contrast agent. Signal intensity of the myocardium can be normalized to a normal skeletal muscle: in this case, a ratio of 4 or more is considered indicative of myocardial inflammation. Alternatively, avoiding normalization, myocardial inflammation may be suggested by signal increase of the myocardium higher than 45% compared to pre-contrast scan.

**Fig. 1:**
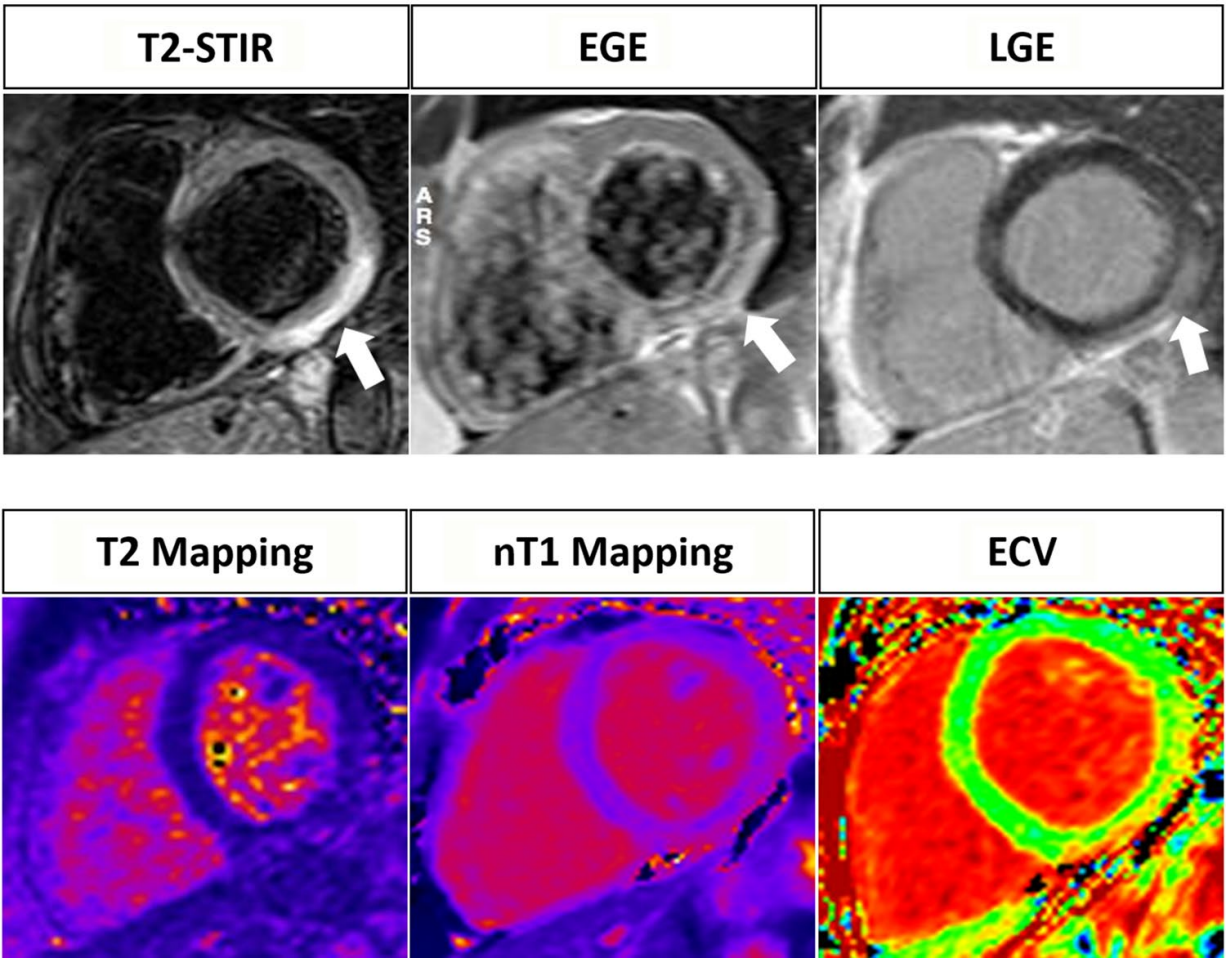
A 57 year old female with sudden onset of retrosternal chest pain. T2-w STIR image shows an hyperintense subepicardial rim representing myocardial edema in the inferior wall of the LV (arrow). EGE image shows an increase of Gd uptake in the same area (arrow). On LGE image an hyperintense area confirming the presence of myocardial necrosis can be observed in the inferior wall of the LV (arrow). The diagnosis of myocarditis can be performed with old LLC (3 criteria out of 3). Mapping images confirm the findings of acute myocardial inflammation: T2 mapping value is higher than 60 ms; nT1 value is higher than 1100 ms and ECV is higher than 32%. Revised LLC are also positive for acute myocardial inflammation (2 criteria out of 2)

Necrosis or fibrosis are both consequences of prolonged or severe tissue damage. Myocyte injury leads to the loss of cell membrane integrity with subsequent necrosis, spill out of debris into the extracellular space, infiltration of inflammatory cells and collagen deposition with development of fibrosis. This mechanism contributes to expand the interstitium, increasing the volume of contrast media in the extracellular space. Necrosis and fibrosis are evaluated using the late gadolinium enhancement (LGE) imaging. LGE images specifically detect expanded extracellular space and they are displayed using inversion recovery prepared gradient echo pulse sequences, which null the normal myocardial signal to zero (Fig. 1).

Mapping techniques, namely native T1 mapping and ECV mapping, are also considered useful tools for the evaluation of fibrosis. The latter is more technically demanding as it requires acquisition of T1 maps pre- and post-administration of contrast media, and adjustment for the hematocrit value. Mapping is complementary to LGE because it enables to detect milder and more diffuse fibrosis.

In the setting of clinically suspected myocarditis, according to the LLC, myocardial inflammation can be diagnosed if at least two out of the three above mentioned CMR criteria are present. Left Ventricular (LV) dysfunction and/or pericardial effusion, common in these patients, are considered ancillary findings.

## Diagnostic accuracy of CMR

The original “Lake Louise Criteria” [13] provided a good overall diagnostic performance, better than any of the individual CMR parameters, and after 10 years of application, their sensitivity, specificity and DA in the clinical suspect of acute myocarditis increased from 67%, 91% and 78–80%, 87% and 84%, respectively [16]. Consequently, they should remain in use in centers that have good experience with their application.

**Fig. 2:**
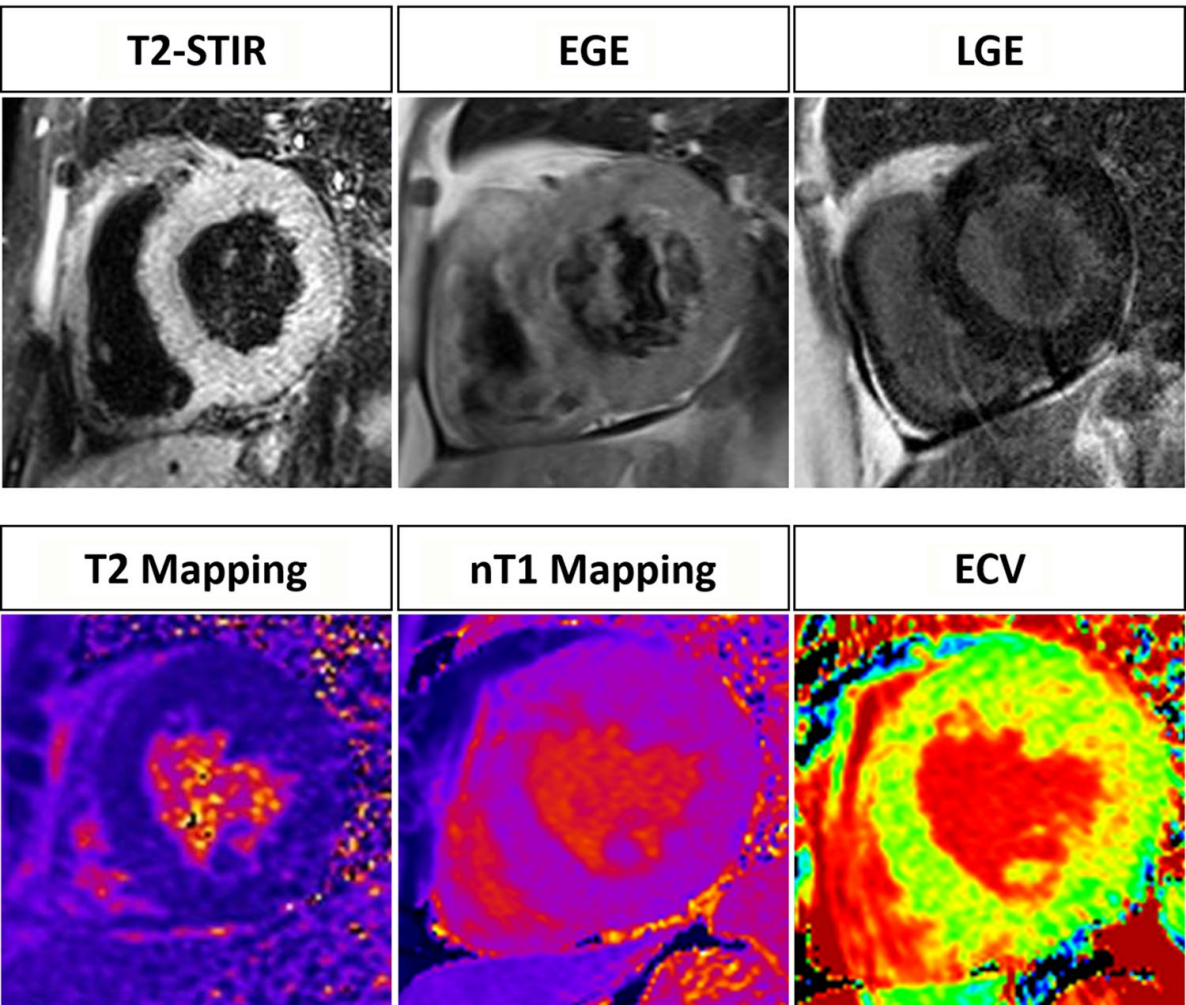
A 55 year old male with malaise and an EF of 33%. No edema, hyperemia and necrosis can be observed in T2-w, EGE and LGE images, respectively. Mapping sequences show an overall value of 58 ms on T2 mapping, an overall value of 1150 ms on nT1 mapping and an overall ECV value of 35%. The diagnosis of acute myocarditis cannot be obtained with the original LLC (0 criteria out of 3), but is provided by applying the revised LLC (2 criteria out of 2)

However, LLC seems to perform better in myocarditis with “infarct-like” presentation compared to cases manifesting with heart failure or arrhythmias (Se = 80% vs 57% and 40%, respectively) [17]. Due to this drawback and to the increasing clinical potential of pixel-wise mapping of T1 and T2 relaxation time, in 2018, Lake Louise Criteria have been updated. With the aim to increase specificity, the rule to define a “positive case” has been slightly modified: the presence of at least one edema-sensitive CMR criteria (T2-weighted images or T2 mapping) combined with at least one additional T1-based tissue characterization technique (LGE, T1 mapping, or ECV) (Fig. 2). Pericardial effusion in Cine CMR images or high signal intensity of the pericardium in LGE, T1 mapping or T2 mapping to detect pericardial inflammation and systolic LV wall motion abnormality in cine CMR to detect LV dysfunction are considered supportive criteria. A great advantage of the revised LLC is a free Gadolinium protocol, when the injection of gadolinium is contraindicated (e.g.,: patients with an history of allergic reaction to gadolinium-based contrast media; pregnant women; patients with end-stage renal insufficiency).

Due to the recent introduction of the revised LLC into clinical practice, to be best of our knowledge there is only one prospective study investigating their diagnostic yield [18]. According to Luetkens and colleagues [18], sensitivity of the revised LLC is significantly higher compared with the sensitivity of the original LLC (*P* = 0.031, 87.5% vs 72.5%). No differences in specificity were observed between the two sets of criteria (*P* = 0.999, 96.2% vs 96.2%). In addition, several relevant information can be extrapolated from a recently published review, considering mapping parameters individually. The pooled weighted specificity, sensitivity and AUC of T2 mapping are 91%, 70% and 0.79, respectively. The pooled weighted specificity, sensitivity and AUC of T1 mapping are 91%, 82% and 0.86 [16]. Authors also concluded that the diagnostic performance of T2 mapping is comparable to the original LLC, while the performance of T1 mapping might be superior. Diagnostic accuracy improves significantly combining parameters two by two, as recently reported by Ferreira and coworkers in a meta-analysis available as supplemental material of the revised LLC paper [19]. T2 mapping and LGE seem to offer the best combination (AUC 0.928; 95%CI 0.811–1.000; heterogeneity 0.382), although this is based on only 2 published studies.

More head-to-head studies comparing conventional CMR with mapping techniques are advisable to define their true diagnostic value.

## Prognosis and follow-up of acute myocarditis

**Fig. 3:**
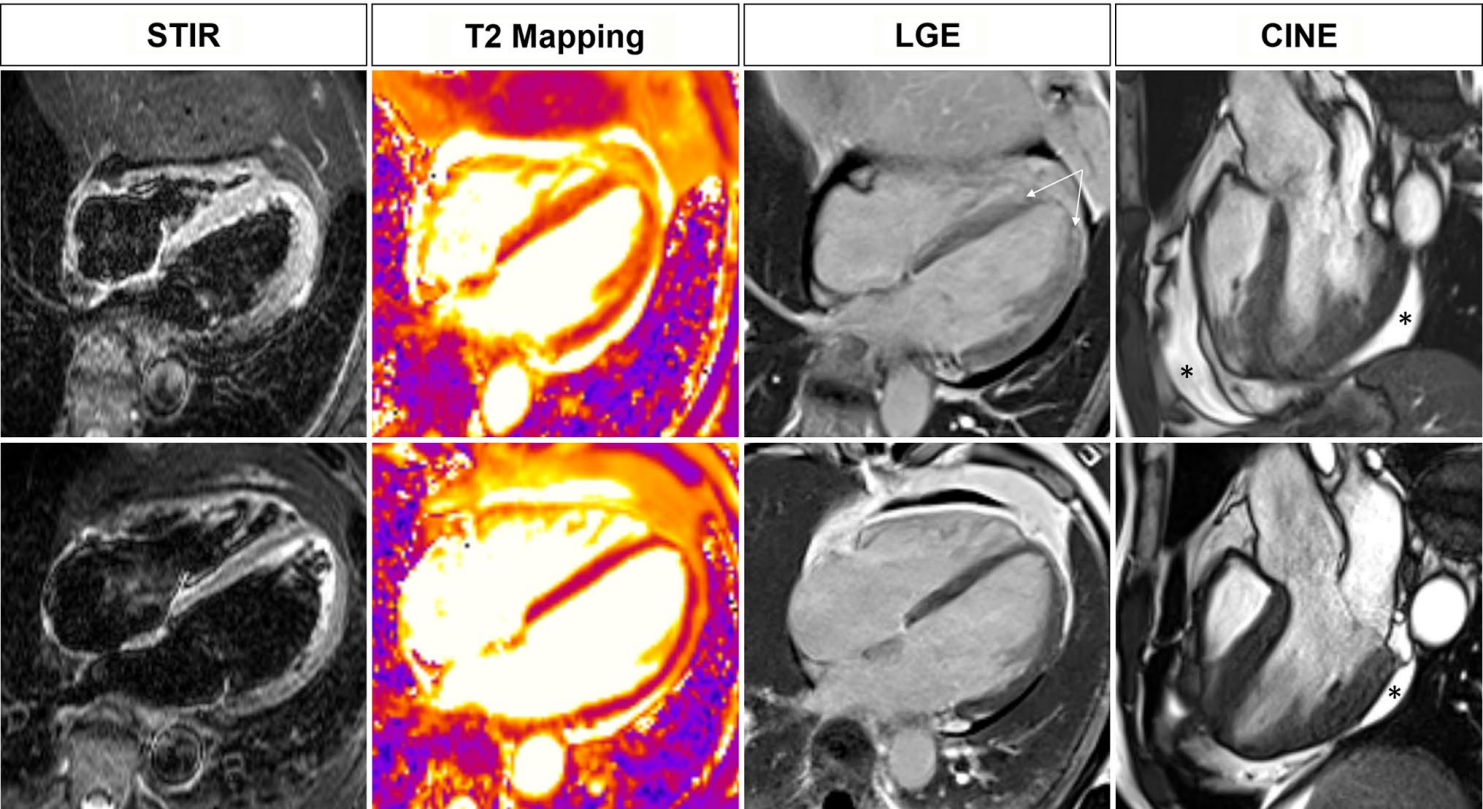
43 year old male with chest pain, and depressed systolic function (20%) during sepsis by Pseudomonas aeruginosa. CMR scan performed in the acute phase (upper row) shows hypersignal of the septum and mid-apical lateral wall, mirrored by prolonged T2 on mapping; these findings are consistent with edema. PSIR shows patchy areas of subepicardial enhancement in the apical segments (arrows). Pericardial effusion (asterisk). Early follow-up scan obtained 30 days later shows normalization of T2 and regression of the enhancement of the myocardium; near complete resolution of the pericardial effusion

In the short- to mid-term, the clinical presentation influences the prognosis of acute myocarditis: recent evidences show that patients presenting with acute fulminant myocarditis—i.e., manifesting heart failure symptoms and hemodynamic compromise requiring pharmacologic or circulatory support—have worse in-hospital prognosis than patients with acute myocarditis—i.e., hemodynamically stable-. This results in a more prolonged observation in intensive care unit for acute fulminant myocarditis [20]. In the long term, acute myocardial inflammation mostly regresses leaving minimal or no functional damage; however, a worse functional outcome is more likely in acute fulminant myocarditis (Fig. 3). The position statement of the European Society of Cardiology suggests the need for long-term follow-up of patients, including those presenting with infarct-like symptoms and no LV functional impairment; timeline and modalities of the follow-up schedule, though, are not specified [5]. Echocardiography provides accurate assessment of LV and right ventricular function [21], but is outperformed by CMR for the evaluation of structural abnormalities of the myocardium.

CMR may play a role in the stratification of the prognosis: two recent meta-analyses proved that LVEF and LGE are strong predictors of major adverse cardiac events (MACE). Cut-off values of LGE (17 grams, or 13% of myocardial mass) [22, 23] were associated with MACE, although such thresholds are not validated for routine application in clinical practice. Noticeably, in a group of 203 patients with biopsy-proven acute myocarditis, none of the patients with LGE had sudden cardiac death in the long term, regardless of LVEF or LV dilatation. LGE persists, although its extent may decrease: it is unclear whether partial regression has clinical significance or not.

Thus far, there are no evidences on the prognostic value of any of the modern quantitative techniques (T2, T1 and ECV mapping). Relaxation times of the myocardium are influenced by a number of factors (vendor, type of sequence, and homogeneity of the magnetic field) that affect reproducibility and comparability of the results in different studies.

## Myocardial inflammation and COVID-19

During the current Sars-CoV2 pandemic several cases of COVID-19 myocarditis were observed, some of which fatal [24]. This was not unexpected: coronavirus is known to induce myocarditis, though not being among the most commonly involved viral agents. Furthermore, cases of myocarditis were reported during the previous outbreaks of SARS and MERS-CoV [25, 26].

Similar to other viral agents, the pathophysiology of COVID-19 myocarditis is the result of both cell infection damage and (auto)immune reaction. Sars-Cov-2 enters human cells binding its spike protein to angiotensin-converting enzyme-2 (ACE-2), which can be found on the membrane of epithelial respiratory cells, cardiomyocytes and pneumocytes (type-2).

Activated T-cells are responsible for cell-mediated cytotoxicity. Noteworthily, cytokine storm, which is known to exacerbate the clinical course of COVID-19, promotes the activation of T-cells, which releasing cytokines maintain the exaggerated immune response.

Cardiomyocyte injury, pericardial inflammation with effusion and microvascular damage may be the substrate of arrhythmia in COVID-19 myocarditis. This might explain some of the sudden cardiac deaths observed in quarantined or discharged patients in northern Italy [27].

**Fig. 4:**
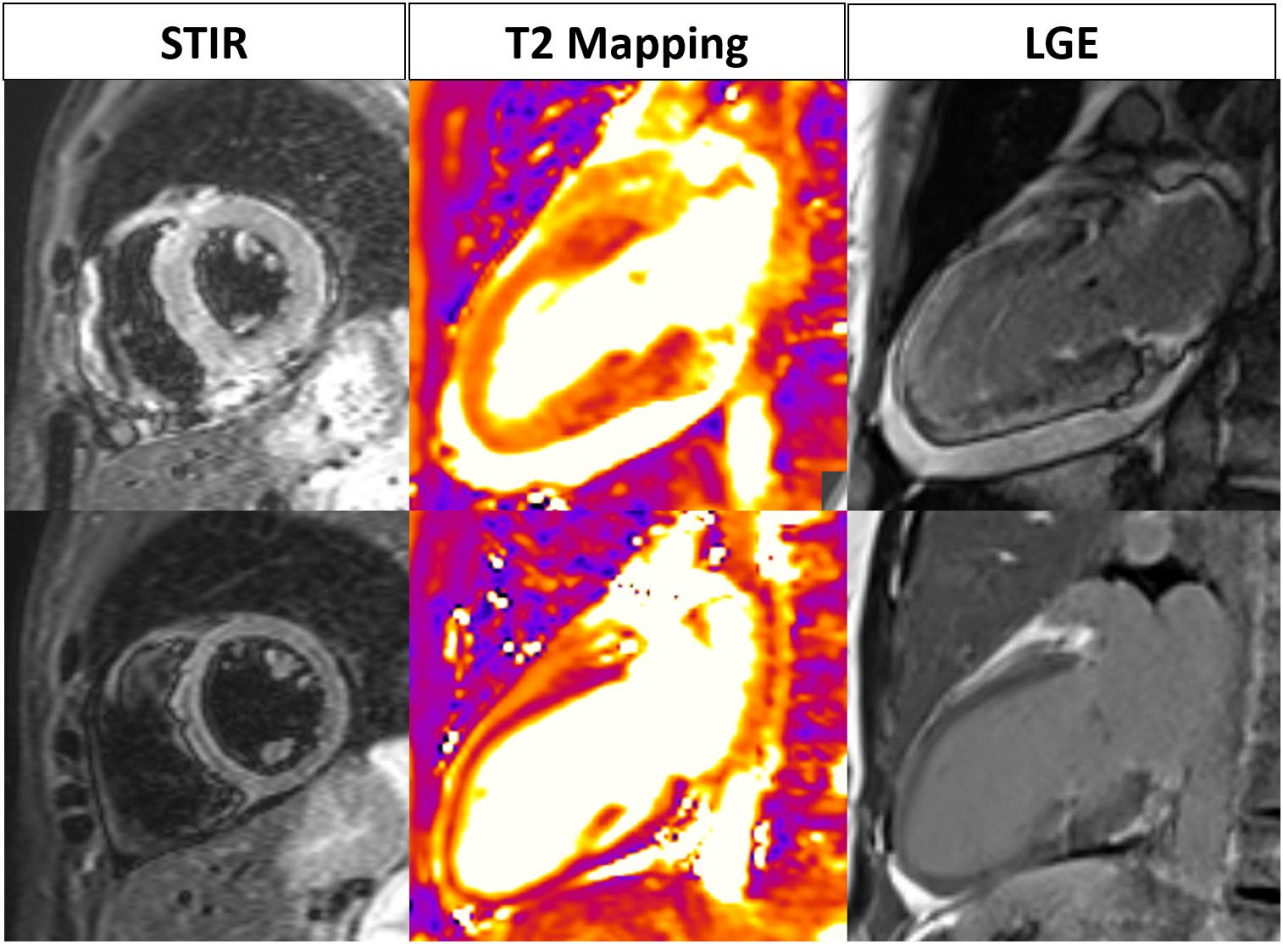
COVID-19 myocarditis in a 54-year old female. In the acute phase (upper row), STIR and T2 mapping depict global edema, resulting in swelling of the myocardium. LGE shows diffuse enhancement with subepicardial gradient. Clinical findings and EF (43%) suggested acute fulminant form. On a follow-up scan (lower row) performed 55 days later near complete regression of radiological findings is documented

CMR findings in COVID-19 related acute myocarditis cases do not differ from what described in Lake Louise criteria [24, 28] (Fig. 4).

## Chronic myocarditis

Chronic immune activation may occur in several conditions, including persistence of viral genome in myocytes, autoimmune diseases eosinophilic syndromes and sarcoidosis and may manifest with organ dysfunction.

Chronic myocarditis tends to occur in older subjects and has more subtle clinical manifestation than the acute form. The onset of symptoms occurs generally more than 30 days prior to presentation and cardiac biomarkers show minimal abnormalities. Typically, LV is dilated with thinned walls.

**Fig. 5:**
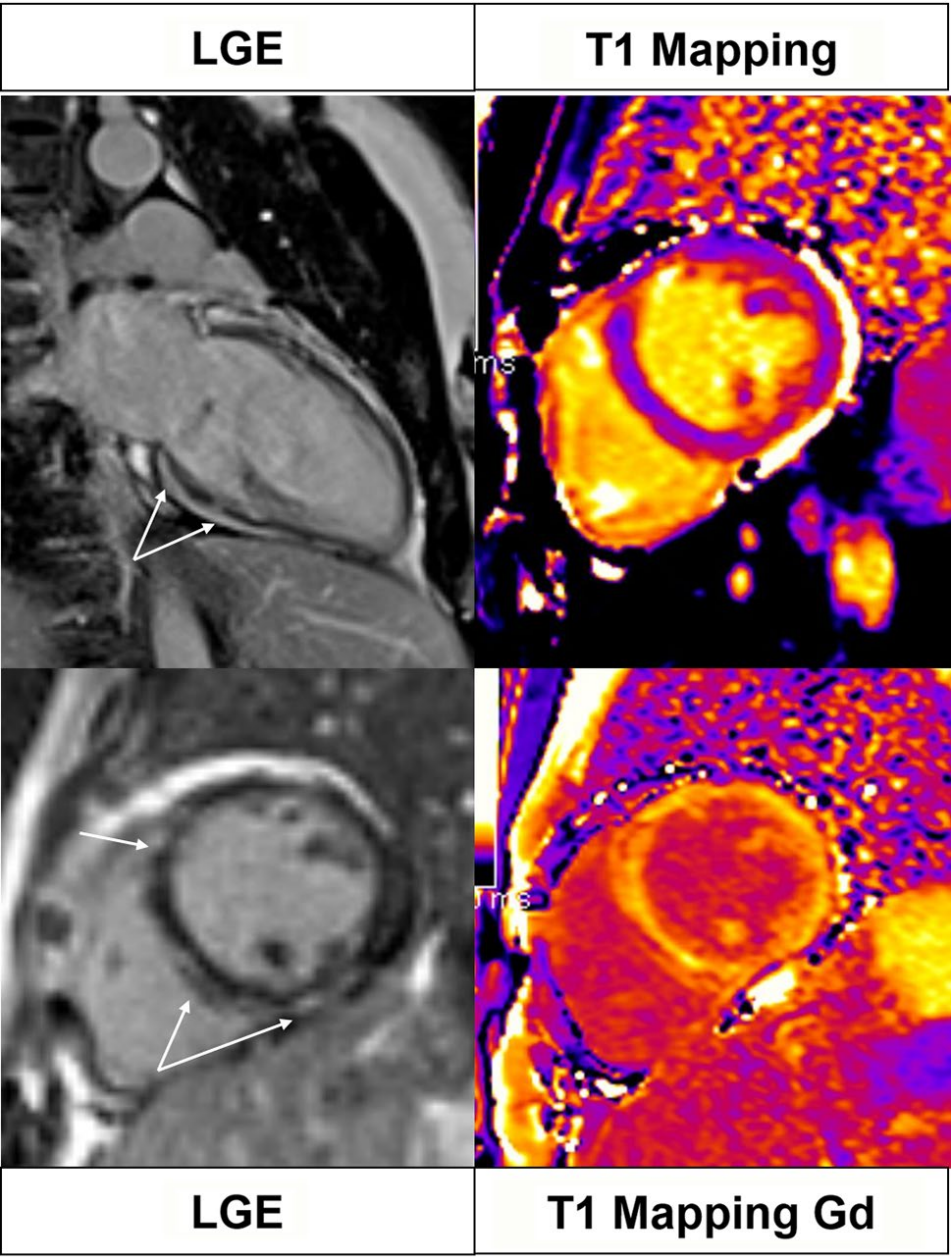
38-year old female with polimorphic extrasystole, regressing on effort, and mild mitral valve regurgitation. Diffuse areas of myocardial enhancement are seen on basal and midcavity inferoseptal and anteroseptal wall, with subepicardial and midwall distribution (arrows). The same segments display focal anomalies of both native and post-contrast T1 mapping. Findings are consistent with chronic myocarditis

In this scenario, the sensitivity of cardiac CMR drops significantly, compared to acute myocarditis. Edema is less prominent and less frequent. Francone et al. [17] observed it in 28% of patients with cardiomyopathic pattern of presentation, as opposed to 81% of patients with infarct-like presentation. The MyoRacer trial [29] found similar incidence of LGE in patients with acute and chronic myocarditis: the latter however had relatively higher intramural enhancement and significantly lower incidence of lateral wall involvement (Fig. 5). Overall, the sensitivity and specificity of CMR for chronic myocarditis are significantly inferior to acute myocarditis (81% and 71% vs 63% and 40%, respectively). There is no data on the performance of the revised LLC in this scenario; however, the MyoRacer trial showed that, in patients with chronic symptoms, T2 mapping was the only sequence able to differentiate patients with acute myocardial inflammation.

## Is there a role for CT in patients with acute and chronic myocarditis?

Clinical presentation of myocarditis is heterogeneous thus the assessment of the coronary arteries is often required to rule out acute coronary syndrome. Multi Detector Computed Tomography (MDCT) plays a pivotal role in this setting, for its low invasiveness combined with an excellent NPV, as high as 99% for significant coronary artery stenosis [30].

As the pharmacokinetics of iodinated and gadolinium-based contrast agents are similar, the technique of late myocardial enhancement could be applied also on MDCT scans [31, 32].

From a technical point of view, CMR has a significant advantage over MDCT, due to the possibility to null the signal of the myocardium and consequently to increase the conspicuity of hyperenhancing areas. On the other hand, MDCT is hampered by low signal-to-noise ratio and contrast resolution.

Decreased tube voltage (70–80 kVp) and increased contrast agent volume may strengthen damaged myocardial density and enhance scar or fibrosis visualization in delayed phase cardiac MDCT at the price of lower signal-to-noise ratio. Furthermore, multi-energy scanners may provide additional improvement of the potential of MDCT. It has been demonstrated that monochromatic images (70–90 keV) with optimal energy levels, derived by multi-energetic acquisition, yield better contrast-to-noise ratio than conventional single-energy polychromatic images commonly used for late enhancement [33].

Similar to CMR, cardiac MDCT images acquired in a delayed phase allow to measure myocardial ECV; the agreement between ECV values provided by these two techniques is good [34–36].

Bouleti and colleagues demonstrated an excellent overall accuracy (95%) of dual energy/spectral CT in the acute myocarditis assessment compared to CMR, in a large population of patients, admitted for chest pain with a final diagnosis of acute myocarditis [37].

CMR is currently the gold standard for the noninvasive diagnosis of myocarditis [4]. From a practical point of view, however, MDCT has several advantages. It is generally more accessible and faster than MRI and permits easier patient monitoring.

**Fig. 6:**
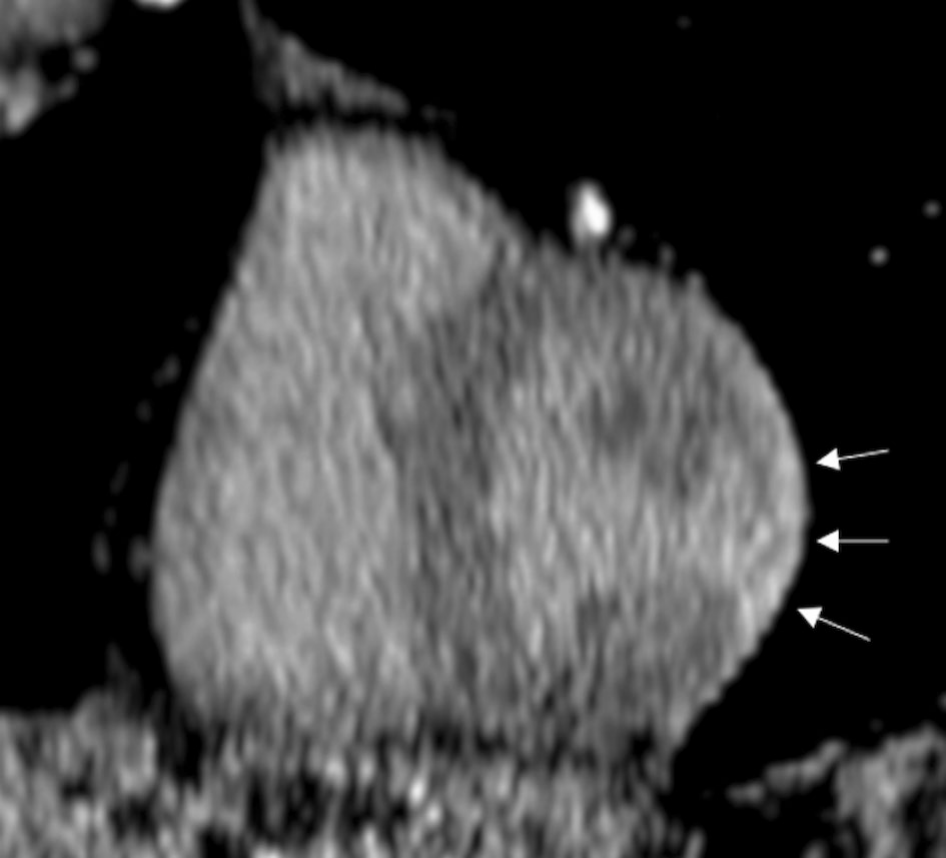
CT examination performed in a patient with chest pain. Late phase acquisition (8 min after contrast administration) using low Kv. Short-axis reformation shows subepicardial enhancement in the mid-ventricular lateral wall, suggestive of myocarditis. The diagnosis was later confirmed by lab test and MRI scan

With optimized late enhancement technique, MDCT could be a promising “one stop shop” exam, alternative to CMR, especially in patients with acute myocarditis with infarct-like presentation (Fig. 6).

## Pericarditis

Pericardial inflammation may occur in a quite varied spectrum of conditions, including infections (viral, bacterial, fungal and tubercular), autoimmune diseases (such as LES, scleroderma, rheumatoid arthritis), primary or secondary pericardial tumors, and chronic kidney disease. Furthermore, pericarditis may be triggered by direct pericardial injury (surgery, radiation therapy on the encompassing the mediastinum) and cardiac damage (transmural infarct and Dressler syndrome). Interestingly, as for chronic myocarditis, viral pericarditis and Dressler syndrome are the result of an immune-mediated damage. Up to 30% of cases have no defined cause and, consequently, are classified as idiopathic.

**Fig. 7:**
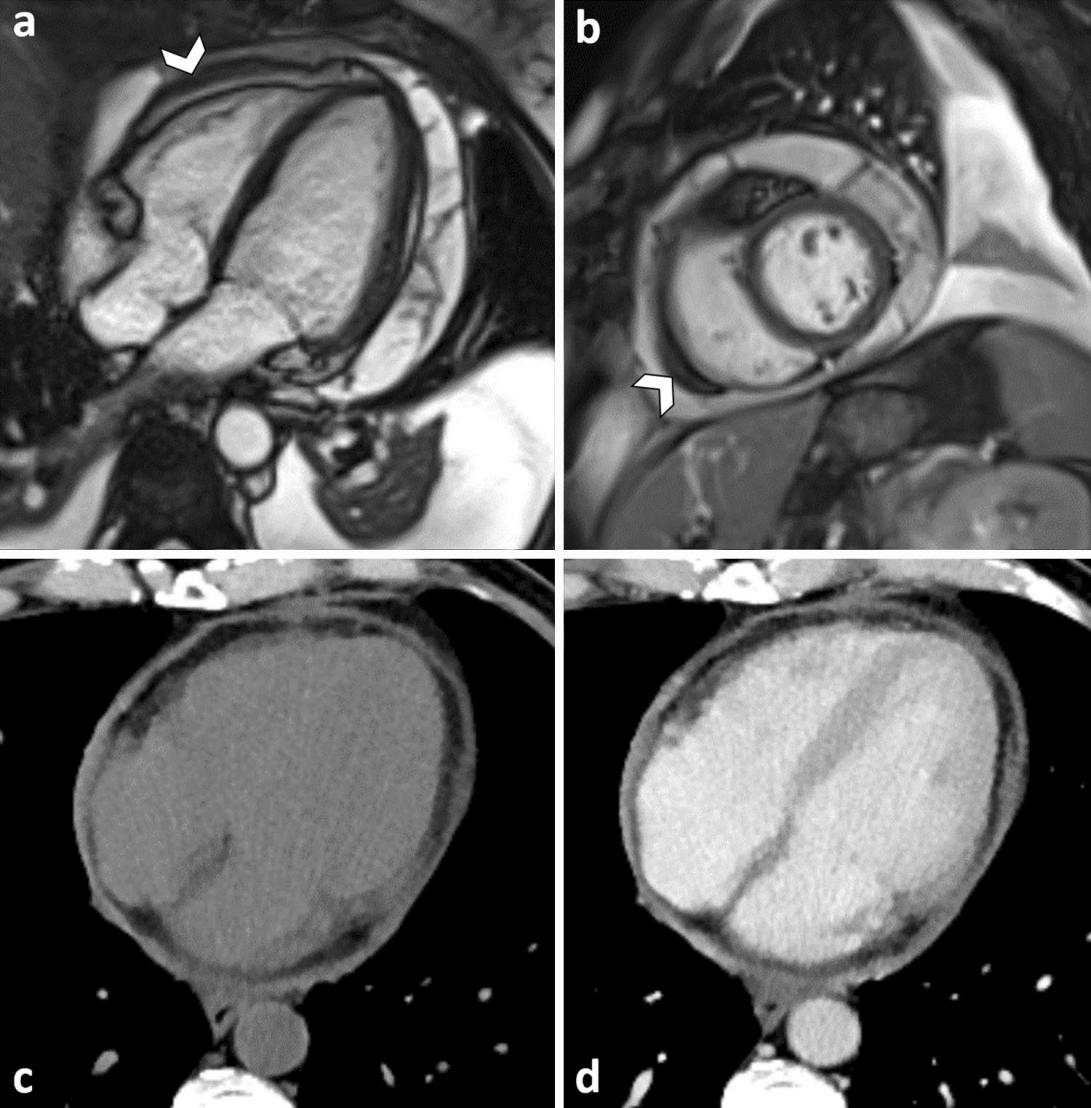
Cine MRI scan on 4-chamber (**a**) and short-axis (**b**) view, shows circumferential pericardial effusion with multiple linear septa within the pericardial sac, indicating fibrin deposition. Note thickening of the visceral pericardium (arrowheads). CT before (**c**) and after contrast (**d**) shows diffuse thickening and enhancement of the pericardium in a patient affected by lung carcinoma

CT and CMR can equally demonstrate pericardial effusion and pericardial thickening: 4 mm thickness is conventionally indicated as the upper limit of normal, although it must be emphasized that pericarditis may be present also when the pericardium is within normal limits. Stranding of the paracardiac fat tissue can be an ancillary finding [38]. CMR better depicts pericardial enhancement (on SE T1 and LGE sequences) and myocardial enhancement: the combination of the two (myopericarditis) entails higher risk of complications [39] (Fig. 7).

In some cases, inflammation causes permanent fibrotic changes and calcium deposition in the pericardium, resulting in constrictive pericarditis. In this condition, the stiffening of the pericardium has effect on ventricular filling: in detail, during inspiration RV filling prevails whereas during expiration LV filling is enhanced. The combination of morphologic changes of the pericardium, remodeling (tubing) of ventricular cavities, biatrial enlargement are indirect signs of constrictive pericarditis. CMR, however, permits direct demonstration of functional alterations: real-time cine sequences acquired during free-breathing show flattening of the interventricular septum at inspiration, followed by return of normal convexity at end expiration (*septal bounce*).

## Conclusion

The protean clinical presentation and varied etiology contribute to make myocarditis a challenging diagnosis, in many cases. Though regarded as the gold standard technique for the diagnosis, EMB is not routinely performed and may be hampered by sampling errors. For these reasons, CMR plays a pivotal role in the evaluation of acute myocardial inflammation. Optimized acquisition protocol, boosted by the application of modern mapping techniques, allows to obtain the diagnosis noninvasively and with very high diagnostic accuracy.
